# Is it possible to extend the dose interval of canakinumab treatment in children with familial Mediterranean fever? PeRA group experience

**DOI:** 10.1186/s12969-023-00925-5

**Published:** 2023-11-23

**Authors:** Gülşah Kavrul Kayaalp, Şengül Çağlayan, Fatma Gül Demirkan, Vafa Guliyeva, Gülçin Otar Yener, Kübra Öztürk, Ferhat Demir, Semanur Özdel, Mustafa Çakan, Hafize Emine Sönmez, Betül Sözeri, Nuray Aktay Ayaz

**Affiliations:** 1 Department of Pediatric Rheumatology, Istanbul Faculty of Medicine, Istanbul University, Istanbul, Turkey; 2 Department of Pediatric Rheumatology, University of Health Sciences, Umraniye Research and Training Hospital, Istanbul, Turkey; 3 Department of Pediatric Rheumatology, Sanliurfa Research and Training Hospital, Sanliurfa, Turkey; 4 Department of Pediatric Rheumatology, Goztepe Prof. Dr. Süleyman Yalçın Research and Training Hospital, Istanbul Medeniyet University, Istanbul, Turkey; 5 Department of Pediatric Rheumatology, Acıbadem Health Groups Hospital, Istanbul, Turkey; 6 Department of Pediatric Rheumatology, University of Health Sciences, Ankara Dr. Sami Ulus Research and Training Hospital, Ankara, Turkey; 7 Department of Pediatric Rheumatology, Kocaeli University Faculty of Medicine, Kocaeli, Turkey

**Keywords:** Familial Mediterranean Fever, Canakinumab, Anti IL-1

## Abstract

**Background:**

There is no clear data on the optimal duration of treatment with anti-interleukin-1 drugs in colchicine-resistant familial Mediterranean fever patients, as well as on the dose interval. This study aimed to assess patients whose canakinumab dose interval was adjusted according to a specific protocol, with the objective of evaluating the effectiveness of implementing this protocol for the patient care.

**Methods:**

The files of 45 patients whose canakinumab treatment interval was opened with a standard protocol previously determined by the Delphi method were retrospectively reviewed.

**Results:**

Canakinumab treatment was initiated once a month for all patients. In the sixth month of canakinumab treatment, a dose interval extension was introduced; however, 7 patients (15.5%) experienced an attack, and consequently, no further interval extension was administered to them. For 29 patients, the dose interval was successfully extended to once every three months, as they remained attack-free for a year after the first interval extension. Nine patients continued receiving the drug every 2 months, as they had not yet completed one year since the first extension. The study found no significant correlation between experiencing an attack during the dose interval extension protocol and the number, duration of attacks, or autoinflammatory diseases activity index score.

**Conclusion:**

Extending treatment intervals with canakinumab in colchicine-resistant familial Mediterranean fever shows promise for favorable outcomes.

**Supplementary Information:**

The online version contains supplementary material available at 10.1186/s12969-023-00925-5.

## Introduction

Familial Mediterranean fever (FMF) is the most common hereditary autoinflammatory syndrome and primarily affects individuals from the Mediterranean basin. The condition manifests as recurrent inflammatory episodes typically marked by fever and serositis [1]. The *MEFV* gene, which is responsible for encoding the pyrin protein, is often mutated in patients with FMF. *MEFV* mutations are known to affect pyrin-mediated regulation of caspase 1 activity in inflammasomes, which can lead to excess production of interleukin-1 (IL-1), a pro-inflammatory cytokine [2].

Colchicine is the recommended treatment and has proven effective in preventing inflammatory attacks as well as the development of amyloidosis, the most important complication associated with this condition [3–5]. However, approximately 5–10% of patients have inadequate response to colchicine [6]. While it is known that colchicine can inhibit IL-1β release from peripheral blood mononuclear cells of FMF patients, the specific efficacy of colchicine in the treatment of FMF and the impact of disease-specific *MEFV* variants on the RhoA-induced pyrin inflammasome and NLRP3 inflammasomes are still unclear [7]. Nevertheless, blocking IL-1β activity has emerged as a target for patients with inadequate response to colchicine, and positive outcomes has been reported [8–12]. However, there is no clear data on the optimal duration of treatment with anti-interleukin-1 drugs, as well as the dose interval. In a Delphi study on the management of FMF patients conducted by our working group ‘PeRA’ in 2020, a consensus was reached on the opening the dose interval of anti-IL-1 therapies. This study suggests that patients who have been free of attacks and subclinical inflammation for the last 6 months after starting biologics can have their treatment intervals extended to twice the original dose intervals. Moreover, for those who remain free of attacks and subclinical inflammation for one year after the extension, treatment intervals can be extended to three times the original dose intervals [13]. The protocol was adopted by centers that reached this consensus in advance and applied to patients receiving canakinumab.

The objective of this study was to assess patients whose canakinumab dose interval was adjusted based on the aforementioned protocol.

## Methods

### Study design and dose interval extension protocol

The study included patients diagnosed with FMF before the age of 18 and receiving canakinumab treatment from seven different centers. All patients met at least one of the Tel-Hashomer or Eurofever/Paediatric Rheumatology International Trials Organisation (PRINTO) 2019 diagnostic criteria for FMF [14, 15].

**Fig. 1:**
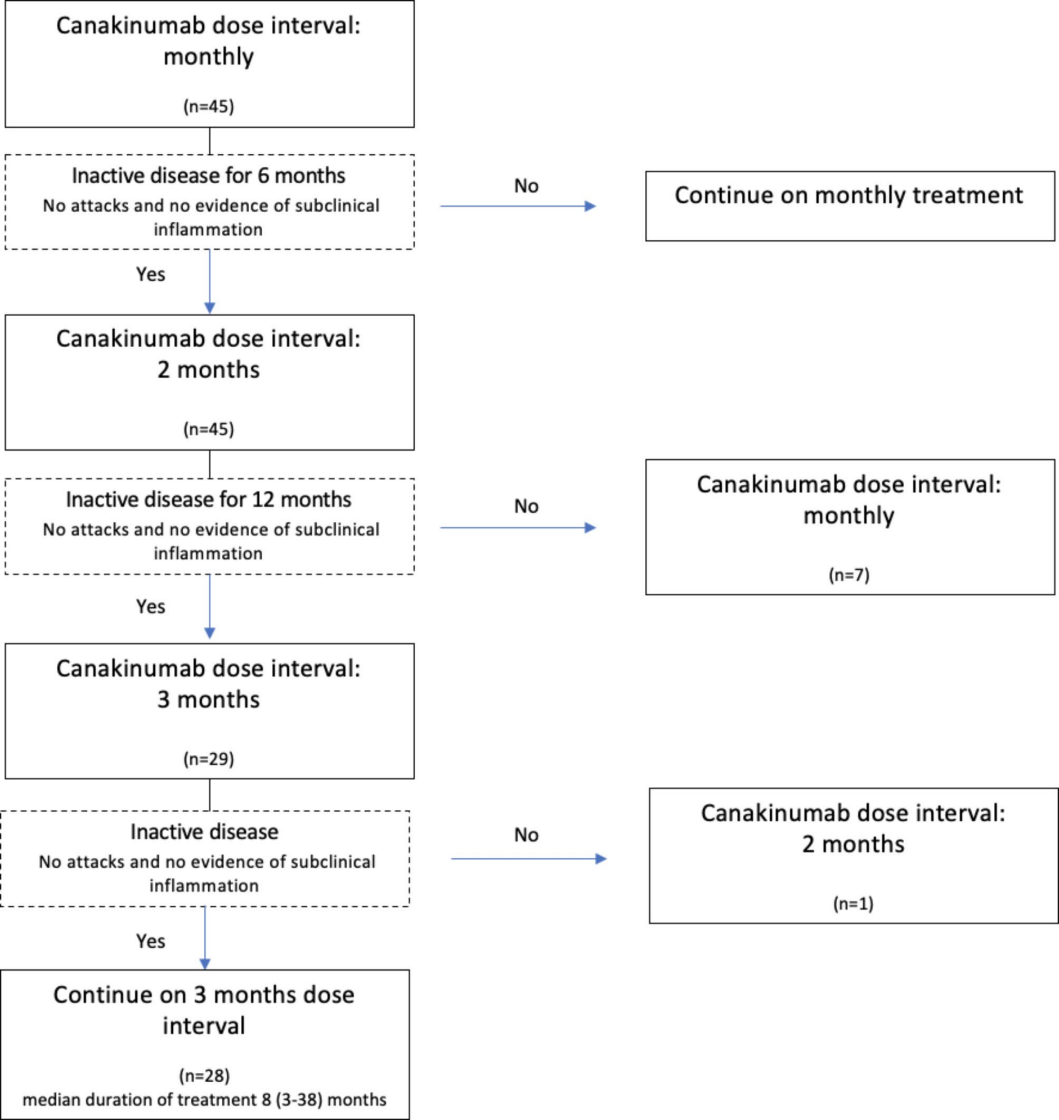
Canakinumab dose interval extension protocol

In order to be included, patients are required to comply with the protocol for extending the dose interval described in the Delphi study. In this Delphi study, recommendations were developed for the diagnosis, follow-up, and colchicine treatment of FMF patients, as well as for determining the dose intervals of anti-IL-1 treatment. According to the Delphi study, a consensus was reached on the dose intervals for anti-IL1 treatment in FMF patients resistant to colchicine: (1) For patients without any attacks and no laboratory evidence of subclinical inflammation within the last 6 months following the initiation of biologics, treatment intervals can be extended to twice the original dose intervals. (2) After the first extension of the treatment intervals, for patients without any attacks and no laboratory evidence of subclinical inflammation within the last 1 year, intervals can be further extended to three times the original dose intervals. In order to evaluate a standard protocol, the first inclusion requirement regarding the canakinumab dose regimen for this study is to initiate canakinumab treatment on a monthly basis and then, after six months, extend the dose interval to two months. The second requirement was that the dosing interval was extended to every three months for patients who were one year after the first dose interval extension and had no subclinical signs of inflammation or a new FMF attack. Patients who did not comply with the mentioned schedule, meaning they did not experience an attack or show any subclinical signs of inflammation, but whose dose range was not extended according to the schedule were excluded from the study. According to protocol, if a patient had at least one attack or showed signs of subclinical inflammation while on the current dosing schedule, the dosing range was not reextended and the previous dosing range was reverted. The diagram of the canakinumab dose interval extension protocol examined in the study is shown in Fig. 1. Furthermore, this dose interval extension protocol is not applied to patients with amyloidosis, therefore patients with amyloidosis were excluded from the study. Informed consent was obtained from all participants. The demographic and baseline clinical characteristics of the patients were recorded, as well as the number of attacks, laboratory values, and autoinflammatory diseases activity index (AIDAI) scores before the initiation of anti-interleukin-1 treatment, and at 6, 18, and 24 months of canakinumab treatment.

FMF attacks were defined as high fever with clinical findings of serositis/arthritis with high C-reactive protein (CRP) level. Subclinical inflammation was defined as elevation of acute phase reactants (CRP, erythrocyte sedimentation rate, or serum amyloid A) between attacks [5, 13, 16]. Colchicine resistance was defined as the presence of six or more attacks per year or ≥ 3 attacks in a 4–6 months period or elevation of two or more of the acute phase reactants in incomplete attacks, or evidence of subclinical inflammation between attacks [13, 17].

### Statistical analysis

Data was collected using Microsoft Excel (Microsoft Corporation, Redmond, WA) and SPSS 17.0 (IBM, Armonk, NY) for the analysis. Descriptive statistics including mean, mode, median, minimum and maximum were conducted according to the distribution of the variables. Categorical variables were compared with the Pearson chi-square test or Fisher’s exact test where appropriate. The Student *t*-test and Mann-Whitney *U* test were used to compare numerical variables. A value of *p* < 0.05 was considered statistically significant.

### Ethics

The study was carried out complied with the Declaration of Helsinki. Approval was obtained for the study protocol from the Ethics Committee of Istanbul University, Istanbul Faculty of Medicine (approved: 21/05/2020/19).

## Results

A total of 45 patients were included in the study. The patient population consisted of 55.6% females and 44.4% males. The median age of the patients was 15.2 (range 3.5–21) years and the median age of diagnosis was 3.66 (range 0.9–17.5) years. The median follow-up period for patients was 90 (24–223) months, and the median follow-up period after initiating canakinumab was 25 (9–56) months. All patients were using colchicine in addition to canakinumab throughout the protocol. The reason for starting biologic therapy was colchicine resistance in all patients. The percentage of patients who received other anti-IL-1 treatments (anakinra) before starting canakinumab was determined to be 51.1% with a median duration of 3 (1–23) months. In all patients, canakinumab treatment was initiated as once a month with a dose of 2–4 mg/kg (maximum 150 mg).

All patients had at least one exon 10 mutation in the *MEFV* gene. None of the patients had amyloidosis or nephrotic proteinuria. Demographic and baseline clinical and laboratory characteristics of the patients who received canakinumab treatment are detailed in Table 1.

**Table 1:** Demographic and baseline characteristics of the study population

*Age of diagnosis (years) *median (min-max)	3.66 (0.9–17.5)
*Current age (years) *median (min-max)	15.2 (3.5–21)
*Follow up duration (months) *median (min-max)	90 (24–223)
*Follow up duration after initiating canakinumab (months) *median (min-max)	25 (9–56)
*Parental consanguinity *n (%)	16 (35.6)
*Family history of FMF *n (%)	30 (66.7)
*Family history of amyloidosis *n (%)	4 (8.9)
*Characteristics during FMF attacks*	
Abdominal pain n (%)	45 (100)
Arthritis n (%)	24 (53.3)
Chest pain n (%)	14 (31.1)
Pericarditis n (%)	3 (6.7)
Fever n (%)	45 (100)
Erysipelas-like erythema n (%)	12 (26.7)
Protracted febrile myalgia n (%)	3 (6.7)
*Laboratory characteristics before initiation of anti IL-1 therapy*	
Hemoglobin (g/dl) median (min-max)	11.8 (8.2–14.6)
Platelet count (10^3^ cells/µl) median (min-max)	311 (139–653)
WBC count (cells/µl) median (min-max)	9700 (3650–18,000)
SAA (mg/L) median (min-max)	127 (1-970)
CRP (mg/L) median (min-max)	32.0 (0.2–346.0)
ESR (mm/h) median (min-max)	27 (4–88)
*Number of attacks in the last 6 months *median (min-max)	6 (2–24)
*Duration of attacks in the last 6 months (day) *median (min-max)	3 (2–5)
*AIDAI score of the last month before biologic treatment *median (min-max)	16 (2–40)
*Anakinra use prior to canakinumab *n (%)	23 (51.1)
*Duration of anakinra use prior to canakinumab (months) *median (min-max)	3 (1–23)
*MEVF sequence variants*	
p.(Met694Val) homozygous n	40
p.(Met694Val)/ p.(Met680Ile) compound heterozygous n	3
p.(Met680Ile)/ p.(Val726Ala) compound heterozygous n	1
p.(Met694Val) heterozygous n	1

AIDAI: Autoinflammatory diseases activity index, CRP: C-reactive protein, ESR: erythrocyte sedimentation rate, FMF: Familial Mediterranean fever, SAA: Serum amyloid A, WBC: White blood cell

**Fig. 2:**
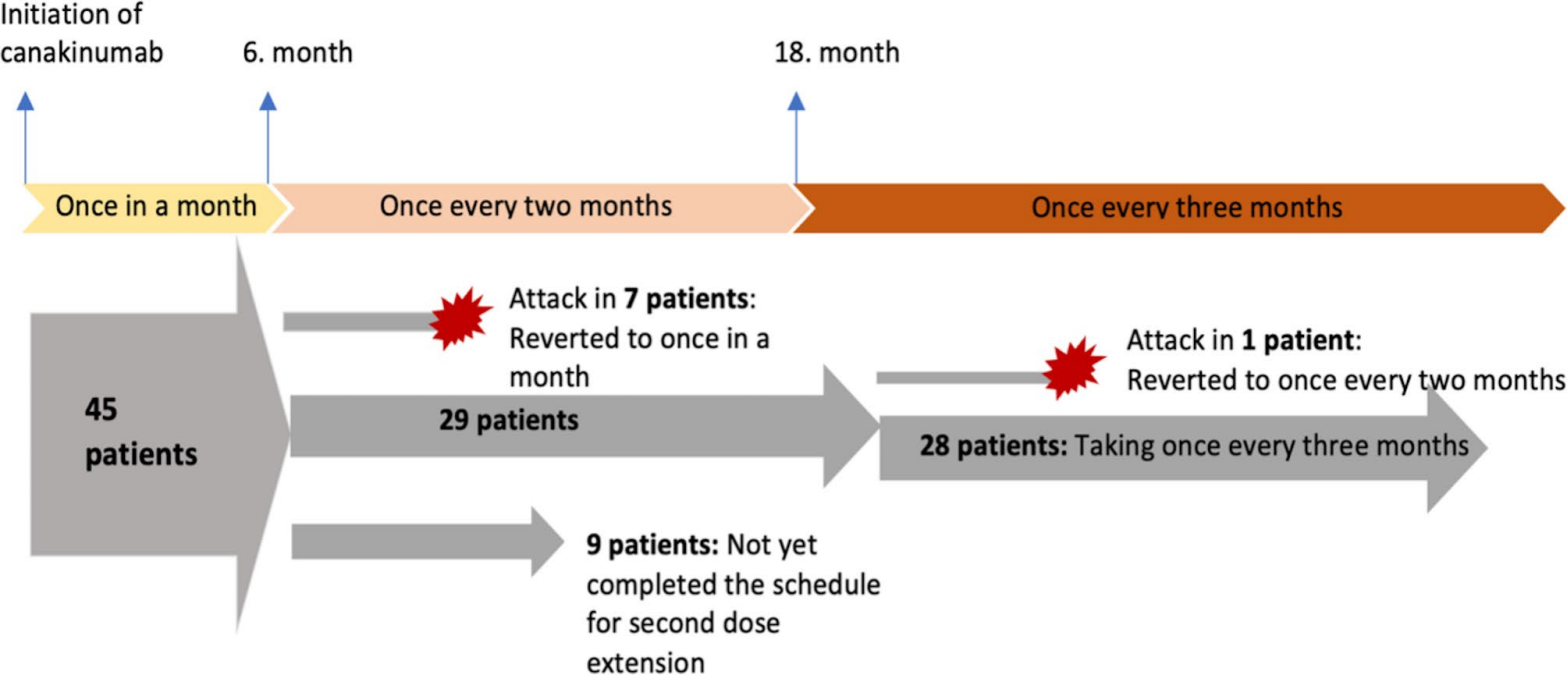
Summary of the patients’ current dosing schedule based on the extension protocol

After six months of receiving canakinumab treatment, 45 patients who showed no evidence of subclinical inflammation and had not experienced any attacks had their dose intervals extended to every two months. Out of the 45 patients, 7 (15.5%) experienced an attack while receiving canakinumab every two months. Consequently, the dose interval was reverted back to once a month and could not be extended again. Out of the remaining 38 patients, 9 were still taking the drug every 2 months because they had not yet completed one year since switching from the previous dosing schedule. The dose interval for 29 patients was extended to once every three months as they had not experienced any attacks for a year, and there were no signs of subclinical inflammation observed during this period. Out of the 29 patients who received canakinumab every three months, one patient experienced an attack on this dosing schedule, therefore the patient’s treatment was switched back to once every two months. The remaining 28 patients (96.6%) continued on the every-three-months schedule, with a median follow-up time of 8 months (range 3–38 months). None of these patients have had any further attacks, and there are no indications of subclinical inflammation. When patients who had prior received anakinra treatment and those who were biologically naive were assessed separately, out of the 23 patients who had used anakinra before transitioning to canakinumab, 16 completed the protocol period, and 11 of them had their dose intervals extended. In the evaluation of 22 biologically naive patients, 18 out of 20 who completed the protocol period were eligible for second dose interval extension. No significant difference was observed between the two groups in terms of achieving a second dose interval extension (Table 2). No side effects related to the use of canakinumab were reported in any patient throughout the entire protocol. A summary of the patients’ condition based on the dose interval extension protocol is provided in Fig. 2. The detailed status of all patients in the extended dose interval protocol are provided in the Supplementary file 1.

**Table 2:** Factors associated with successful transition to the 3-month dosing interval in patients treated with canakinumab

	Dose interval extended to 1 in 3 months (n = 29)	Dose interval cannot be extended to 1 in 3 months (n = 7)	P value
Abdominal pain n (%)	29 (100)	7 (100)	n/a
Arthritis n (%)	15 (51.7)	3 (42.9)	1.00 ^*f*^
Chest pain n (%)	8 (27.6)	4 (57.1)	0.190 ^*f*^
Pericarditis n (%)	3 (10.3)	0	1.00 ^*f*^
Fever n (%)	29 (100)	7 (100)	n/a
Erysipelas-like erythema n (%)	6 (20.7)	2 (28.6)	0.639 ^*f*^
Protracted febrile myalgia n (%)	2 (6.9)	1 (14.3)	0.488 ^*f*^
Anakinra use prior to canakinumab n (%)	11 (37.9)	5 (71.4)	0.204 ^*f*^
Anemia n (%)	9 (31.0)	4 (57.1)	0.225 ^*f*^
Splenomegaly n (%)	3 (10.3)	3 (42.9)	0.073 ^*f*^
Age at diagnosis (years) median (min-max)	3.5 (0.92-14.0)	3 (1.92–9.67)	0.549
Number of attacks in the last 6 months median (min-max)	6 (2–24)	5 (3–24)	0.762
Duration of attacks in the last 6 months (day) median (min-max)	3 (2–5)	3 (2–5)	0.902
AIDAI score of the last month before biologic treatment median (min-max)	18 (2–40)	16 (2–40)	0.564
WBC count (cells/µl) median (min-max)	9500 (4600–18,000)	10,760 (6100–15,510)	0.766
Hemoglobin (g/dl) median (min-max)	12.0 (10.3–14.6)	11.0 (8.9–13.2)	0.166
Platelet count (10^3^ cells/µl) median (min-max)	298 (145–620)	317 (190–653)	0.655
ESR (mm/h) median (min-max))	27 (4–63)	22 (8–88)	0.609
SAA (mg/L) median (min-max)	71.5 (1.0-970.0)	55.0 (13.0-191.0)	0.877
CRP (mg/L) median (min-max)	42.0 (0.2–346.0)	56 (18–214)	0.418
Duration of anakinra use prior to canakinumab(months) median (min-max)	4.0 (1–23)	3.0 (1–6)	0.254
Colchicine treatment duration prior to canakinumab (months) median (min-max)	81.0 (4-192)	49.0 (6-125)	0.401

AIDAI: Autoinflammatory diseases activity index, CRP: C-reactive protein, ESR: erythrocyte sedimentation rate, SAA: Serum amyloid A, WBC: White blood cell

^*f*^ Fisher’s exact test

There was no correlation between failure to extend the dose interval to three months due to an attack and age, age at diagnosis, number of attacks, duration of attacks, AIDAI score, laboratory values, prior use of other anti-IL-1 agents before canakinumab, duration of use of colchicine or other anti IL-1 agents, and clinical findings during an attack (Table 2).

## Discussion

In this study, patients with familial Mediterranean fever who were treated with canakinumab and whose dose intervals were adjusted according to a standard protocol were evaluated. The study focused only on patients who followed the protocol, demonstrating that it is possible to successfully integrate this protocol with canakinumab therapy. Our study represents the largest number of patient evaluation of canakinumab dose interval extension using a standardized protocol. It is worth noting that the protocol used in this study was developed using the Delphi method, a consensus-building approach that involved pediatric rheumatologists from multiple centers, and this protocol has been implemented in their clinical practice [13, 18].

The use of anti-interleukin-1 therapies in patients with colchicine-resistant FMF is a relatively new concept that has gained significant attention in recent years. Currently, guidelines recommend the use of anti-IL-1 therapy for patients who do not respond adequately to colchicine [5, 17]. Randomized controlled studies have demonstrated the efficacy of canakinumab in managing and preventing flares of familial Mediterranean fever in children. Overall, the treatment is considered safe, with the most frequently reported side effects being mild infections, abdominal pain, headaches, and injection-site reactions. Serious side effects, on the other hand, have been rarely observed [10, 12, 19]. However, data on anti-IL-1 treatment duration are limited to case series, and there are no clear recommendations regarding treatment duration [5, 17]. Interestingly, in clinical practice, it has been observed that attacks do not recur after discontinuation of anti-IL-1 therapy in some patients. This phenomenon may be explained by the termination of the ‘autonomous’ state in patients with IL-1 blockade. An autonomous state within inflammatory diseases is related to a constitutively active and self-amplifying innate immune response, which is most prominently observed in individuals with cryopyrin-associated periodic syndrome (CAPS). Nonetheless, in some individuals with FMF, it is suggested that unexpectedly prolonged episodes or frequent recurrent attacks may be attributed to a vicious circle of pro-inflammatory cytokine production triggered by a stressful condition. The administration of biologic agents targeting IL-1 may interrupt this autonomous IL-1β production in some colchicine-refractory FMF patients, leading to a more stable disease course and a restored positive response to colchicine treatment [20]. Nonetheless, there are still only a limited number of studies on the cessation of canakinumab treatment in FMF patients.

In the randomized controlled CLUSTER trial evaluating the efficacy and safety of canakinumab, patients who achieved remission at week 16 had their dose interval extended to once every 8 weeks. The study found that an extended dosing interval of canakinumab every 8 weeks was effective in maintaining disease control in 46% of patients with colchicine-resistant FMF [12]. In the open-label extension of the study, it was found that 53.2% of the patients who received canakinumab once every 8 weeks were able to maintain the same dose and complete the 72-week follow-up period [21]. In another study, canakinumab treatment was initiated at baseline with a frequency of every 2 months for all patients. The study reported that if patients remained attack-free for at least 6 months, the treatment interval was extended to once every 3 months. At the last visit, 4 out of 28 patients were receiving canakinumab every 3 months. However, the study did not provide detailed information on the process of dose interval extension [22]. A more recent study retrospectively evaluated extending the canakinumab dosing interval in 58 pediatric patients undergoing various dose interval extension schedules. The interval was extended for a median of 6 months (3–18 months) after initiation of therapy, and among these patients, the interval was subsequently decreased in four cases due to an attack. The study also found that canakinumab was withdrawn in 12 patients, and among those patients, two experienced an attack after discontinuation of treatment [23]. In a study that evaluated a standard protocol for extending the canakinumab interval, the dose interval was increased to once every 2 months 6 months after the start of canakinumab treatment, and discontinued after the next 6 months. However, the sample size in this study was small, with only 7 patients completing the protocol. Among the 7 patients, 4 experienced relapses after the discontinuation of treatment [24]. In a retrospective study involving adult FMF patients examining the tapering and discontinuation of canakinumab, 22 out of 57 patients receiving canakinumab monthly had their dosing interval extended to 8–12 weeks after 6 months. Among these patients, 12 discontinued canakinumab after a 6-month attack-free follow-up period. It was noted that treatment was reinstated in 3 patients who had initially discontinued due to re-attacks, while the other 9 patients remained in remission with colchicine alone [25]. In a more recent pediatric study, researchers implemented a predefined schedule to discontinue canakinumab treatment in 25 colchicine-resistant FMF patients. Patients with clinically inactive disease adhered to the schedule, which involved doubling the dose interval at 6 months, tripling it at 12 months, and ultimately discontinuing treatment at 18 months. After the completion of the 18-month period, canakinumab treatment was discontinued in 18 out of the 25 patients (72%). A comparison was conducted with patients who did not follow a standardized protocol, and the results showed no significant difference in relapse rates between the two groups. The compared group in this study lacked a standardized protocol, leaving the schedule and dose intervals for each patient unspecified. Based on these findings, the authors concluded that implementing a standardized protocol for ceasing canakinumab treatment can be an effective approach, enabling the potential reduction of treatment duration [26].

Our study demonstrated the efficacy of the canakinumab dose interval extension protocol in a large series of pediatric patients. The dose interval was effectively extended to once every 3 months in 28 patients. The fact that success of dose interval extension was not associated with disease severity, attack frequency before treatment, acute phase marker levels, or disease duration indicates that this dose interval extension protocol can be considered in patients regardless of these factors. In fact, our study showed that even patients who had 24 attacks in 6 months prior to biologic treatment were able to successfully extend the interval to 1 in 3 months (Supplementary file 1). The study also demonstrated that patients who successfully tolerated switching from a monthly dose to once every 2 months also tolerated further extension to a 3-month interval. Hence, it seems reasonable to consider repeating the dose interval extension in patients who remain attack-free while receiving the drug every 2 months.

While our study offers valuable insights into the efficacy of the dose interval extension protocol, there are some limitations to our findings. Specifically, our study did not provide information on the discontinuation of the drug after the dosing interval was increased, and we lacked data on the follow-up period after the drug was stopped. Additionally, due to the retrospective design of our study, not all patients completed the full treatment schedule, as some had not yet reached the second dose interval extension point. Therefore, we are planning to provide long-term results of our protocol to further investigate the efficacy of the protocol. Another limitation of our study is the small number of patients who were unable to undergo a second dose interval extension, which may have limited our ability to thoroughly investigate the factors that influence the success of a second extension. For a more comprehensive understanding of the long-term effects of the protocol, it is essential to conduct larger and global multicenter clinical studies with extended follow-up periods.

## Conclusion

This study suggests the feasibility of the protocol designed to extend the dosing interval of canakinumab. It demonstrated that patients who tolerated the first dose interval extension also exhibit good tolerance to subsequent dose interval extensions. Nonetheless, further studies incorporating additional data are warranted to ascertain the optimal dosing intervals and cessation strategies for this patient population.

### Electronic supplementary material

Below is the link to the electronic supplementary material.

Supplementary Material 1: The status of all patients in the extended dose interval protocol.

## Data Availability

The datasets generated during and/or analysed during the current study are available from the corresponding author on reasonable request.

## Abbreviations

AIDAI: Autoinflammatory diseases activity index
FMF: Familial Mediterranean Fever
IL-1: Interleukin-1
PRINTO: Paediatric Rheumatology International Trials Organisation
