# Supplementary material for: Is it possible to extend the dose interval of canakinumab treatment in children with familial Mediterranean fever? PeRA group experience
Source: Pediatr Rheumatol Online J. 2023 Nov 23;21:140. doi: 10.1186/s12969-023-00925-5 (PMC10666318; doi:10.1186/s12969-023-00925-5)
Supplement: Supplementary file 1 — Supplementary Material 1: The status of all patients in the extended dose interval protocol. [file 12969_2023_925_MOESM1_ESM.docx]

**Article title:** **IS IT POSSIBLE TO EXTEND THE DOSE INTERVAL OF CANAKINUMAB TREATMENT IN CHILDREN WITH FAMILIAL MEDITERRANEAN FEVER?: PERA GROUP EXPERIENCE**

**Journal name: “Pediatric Rheumatology”**

Gülşah Kavrul Kayaalp, Şengül Çağlayan, Fatma Gül Demirkan, Vafa Guliyeva, Gülçin Otar Yener, Kübra Öztürk, Ferhat Demir, Semanur Özdel, Mustafa Çakan, Hafize Emine Sönmez, Betül Sözeri, Nuray Aktay Ayaz, for the PeRA Research Group

Affiliation of the corresponding author: Department of Pediatric Rheumatology, , Istanbul University, Istanbul Faculty of Medicine, Istanbul, Turkey

e-mail address of the corresponding author: [nurayaktay@gmail.com](mailto:nurayaktay@gmail.com)

Funding: No funding was received.

Authors declare no conflicts of interest.

**Supplementary table.** The status of all patients in the extended dose interval protocol

| Patient number | Follow-up duration  (months) | Follow-up duration after CAN  (months) | Number of attacks in the last 6 months* | AIDAI score of the last month* | Anakinra use ** | Duration of anakinra use**  (months) | First dose extension in 6th month | Outcome after the first dose extention | Second dose extention in 18th month | Outcome after the second dose extention | Follow-up duration after the second dose extention (months) | Current status |
| --- | --- | --- | --- | --- | --- | --- | --- | --- | --- | --- | --- | --- |
| 1 | 90 | 27 | 12 | 36 | yes | 2 | yes | No attacks or subclinical inflammation | yes | No attacks or subclinical inflammation | 9 | 3-month intervals |
| 2 | 211 | 21 | 8 | 9 | yes | 7 | yes | No attacks or subclinical inflammation | yes | No attacks or subclinical inflammation | 3 | 3-month intervals |
| 3 | 43 | 25 | 5 | 12 | yes | 1 | yes | Attack observed, dose reverted to monthly | no | - | - | monthly |
| 4 | 103 | 30 | 2 | 10 | yes | 23 | yes | No attacks or subclinical inflammation | yes | No attacks or subclinical inflammation | 12 | 3-month intervals |
| 5 | 79 | 30 | 3 | 8 | yes | 1 | yes | Attack observed, dose reverted to monthly | no | - | - | monthly |
| 6 | 151 | 23 | 4 | 12 | yes | 1 | yes | No attacks or subclinical inflammation | yes | No attacks or subclinical inflammation | 5 | 3-month intervals |
| 7 | 199 | 29 | 3 | 30 | no | - | yes | No attacks or subclinical inflammation | yes | Attack observed, dose reverted to every 2 months | - | 2-month intervals |
| 8 | 139 | 25 | 5 | 24 | no | - | yes | No attacks or subclinical inflammation | yes | No attacks or subclinical inflammation | 7 | 3-month intervals |
| 9 | 58 | 23 | 6 | 30 | yes | 8 | yes | No attacks or subclinical inflammation | yes | No attacks or subclinical inflammation | 5 | 3-month intervals |
| 10 | 175 | 31 | 2 | 15 | no | - | yes | No attacks or subclinical inflammation | yes | No attacks or subclinical inflammation | 13 | 3-month intervals |
| 11 | 223 | 31 | 4 | 36 | no | - | yes | No attacks or subclinical inflammation | yes | No attacks or subclinical inflammation | 13 | 3-month intervals |
| 12 | 59 | 26 | 3 | 18 | no | - | yes | No attacks or subclinical inflammation | yes | No attacks or subclinical inflammation | 8 | 3-month intervals |
| 13 | 47 | 25 | 8 | 40 | yes | 4 | yes | No attacks or subclinical inflammation | yes | No attacks or subclinical inflammation | 7 | 3-month intervals |
| 14 | 103 | 22 | 8 | 30 | yes | 5 | yes | No attacks or subclinical inflammation | yes | No attacks or subclinical inflammation | 4 | 3-month intervals |
| 15 | 40 | 14 | 5 | 30 | no | - | yes | No attacks or subclinical inflammation | Not yet completed the schedule | - | - | 2-month intervals |
| 16 | 64 | 14 | 2 | 18 | no | - | yes | No attacks or subclinical inflammation | Not yet completed the schedule | - | - | 2-month intervals |
| 17 | 103 | 17 | 3 | 30 | yes | 1 | yes | No attacks or subclinical inflammation | Not yet completed the schedule | - | - | 2-month intervals |
| 18 | 146 | 21 | 5 | 40 | yes | 6 | yes | Attack observed, dose reverted to monthly | no | - | - | monthly |
| 19 | 79 | 17 | 5 | 10 | yes | 9 | yes | No attacks or subclinical inflammation | Not yet completed the schedule | - | - | 2-month intervals |
| 20 | 52 | 31 | 4 | 32 | no | - | yes | No attacks or subclinical inflammation | yes | No attacks or subclinical inflammation | 13 | 3-month intervals |
| 21 | 32 | 23 | 12 | 12 | no | - | yes | No attacks or subclinical inflammation | yes | No attacks or subclinical inflammation | 5 | 3-month intervals |
| 22 | 132 | 25 | 12 | 18 | no | - | yes | No attacks or subclinical inflammation | yes | No attacks or subclinical inflammation | 7 | 3-month intervals |
| 23 | 24 | 18 | 18 | 16 | no | - | yes | Attack observed, dose reverted to monthly | no | - | - | monthly |
| 24 | 36 | 24 | 12 | 18 | no | - | yes | No attacks or subclinical inflammation | yes | No attacks or subclinical inflammation | 6 | 3-month intervals |
| 25 | 167 | 56 | 8 | 2 | no | - | yes | No attacks or subclinical inflammation | yes | No attacks or subclinical inflammation | 38 | 3-month intervals |
| 26 | 99 | 42 | 9 | 3 | yes | 10 | yes | No attacks or subclinical inflammation | yes | No attacks or subclinical inflammation | 24 | 3-month intervals |
| 27 | 48 | 25 | 4 | 2 | no | - | yes | Attack observed, dose reverted to monthly | no | - | - | monthly |
| 28 | 90 | 9 | 14 | 7 | yes | 3 | yes | No attacks or subclinical inflammation | Not yet completed the schedule | - | - | 2-month intervals |
| 29 | 36 | 12 | 24 | 12 | yes | 3 | yes | No attacks or subclinical inflammation | Not yet completed the schedule | - | - | 2-month intervals |
| 30 | 66 | 11 | 18 | 9 | yes | 2 | yes | No attacks or subclinical inflammation | Not yet completed the schedule | - | - | 2-month intervals |
| 31 | 89 | 11 | 15 | 9 | yes | 1 | yes | No attacks or subclinical inflammation | Not yet completed the schedule | - | - | 2-month intervals |
| 32 | 25 | 12 | 12 | 8 | yes | 3 | yes | No attacks or subclinical inflammation | Not yet completed the schedule | - | - | 2-month intervals |
| 33 | 132 | 15 | 24 | 21 | yes | 3 | yes | Attack observed, dose reverted to monthly | no | - | - | monthly |
| 34 | 84 | 10 | 24 | 20 | yes | 4 | yes | Attack observed, dose reverted to monthly | no | - | - | monthly |
| 35 | 120 | 25 | 12 | 19 | yes | 3 | yes | No attacks or subclinical inflammation | yes | No attacks or subclinical inflammation | 7 | 3-month intervals |
| 36 | 101 | 28 | 6 | 15 | no | - | yes | No attacks or subclinical inflammation | yes | No attacks or subclinical inflammation | 10 | 3-month intervals |
| 37 | 31 | 27 | 24 | 36 | no | - | yes | No attacks or subclinical inflammation | yes | No attacks or subclinical inflammation | 9 | 3-month intervals |
| 38 | 137 | 27 | 24 | 36 | no | - | yes | No attacks or subclinical inflammation | yes | No attacks or subclinical inflammation | 9 | 3-month intervals |
| 39 | 38 | 27 | 6 | 12 | no | - | yes | No attacks or subclinical inflammation | yes | No attacks or subclinical inflammation | 9 | 3-month intervals |
| 40 | 68 | 26 | 6 | 15 | no | - | yes | No attacks or subclinical inflammation | yes | No attacks or subclinical inflammation | 8 | 3-month intervals |
| 41 | 44 | 26 | 6 | 12 | no | - | yes | No attacks or subclinical inflammation | yes | No attacks or subclinical inflammation | 8 | 3-month intervals |
| 42 | 130 | 26 | 12 | 15 | no | - | yes | No attacks or subclinical inflammation | yes | No attacks or subclinical inflammation | 8 | 3-month intervals |
| 43 | 164 | 26 | 6 | 18 | no | - | yes | No attacks or subclinical inflammation | yes | No attacks or subclinical inflammation | 8 | 3-month intervals |
| 44 | 124 | 21 | 6 | -*** | yes | 3 | yes | No attacks or subclinical inflammation | yes | No attacks or subclinical inflammation | 3 | 3-month intervals |
| 45 | 152 | 21 | 4 | -*** | yes | 2 | yes | No attacks or subclinical inflammation | yes | No attacks or subclinical inflammation | 3 | 3-month intervals |

*prior to biologic treatment

**prior to canakinumab

***missing data

CAN: canakinumab
